# Recurrent locked knee caused by an impaction fracture following inferior patellar dislocation: a case report

**DOI:** 10.1186/1752-1947-5-347

**Published:** 2011-08-03

**Authors:** David Barlow, Keen S Foong, Shin J Rhee, William Sutcliffe, Stuart J Griffin

**Affiliations:** 1 Department of Orthopaedics, Ysbyty Gwynedd Hospital, North Wales, UK

## Abstract

**Introduction:**

Locked knee caused by inferior patellar dislocation is considered rare in elderly patients. It was originally thought that, in the osteoarthritic knee, osteophytes on the pole of the patella become entrapped in the inter-condylar notch, which is managed by performing closed reduction and immobilization in a knee splint for three to four weeks. We present an unusual case of a locked knee with an impaction fracture. To the best of our knowledge, there have been no previous reports of such impaction fractures managed with arthroscopy.

**Case presentation:**

We present an unusual case of an 88-year-old Caucasian woman with moderate arthritis who had a locked knee caused by an impaction fracture of the patella into the lateral femoral condyle. In this case report, we describe the need for arthroscopic surgery to prevent relocking of the knee in these patients.

**Conclusions:**

This case report emphasizes the need for careful assessment of locked knees in elderly patients. Impaction fractures should be considered in all rare cases of patellar dislocation, and we advocate arthroscopic assessment of the articular cartilage in these patients. This is an important consideration, as the population demographics change and such impaction fractures may become more common in patients with degeneration in the knees.

## Introduction

Patients with locked knees present to orthopedic and emergency departments relatively often, and the many causes of this entity are well documented in the literature [1,2]. These include meniscal lesions, loose bodies, ligament injuries, hematomas, tumors, and patellar dislocations [3-5].

Locked knee presenting with inferior patellar and intra-articular dislocations is considered less common in elderly patients and is thought to be the result of osteophytes on the pole of the patella that become entrapped in the inter-condylar notch. Earlier reports have recommended simple manipulation in elderly patient with degenerative knee disease, followed by three to four weeks of support in a knee splint [6-9]. More recently, Syed and Ramesh [10] reported this mechanism of knee locking in an elderly patient who required an open operative procedure to prevent relocking. Their article also described damage to the femoral condyle. In 2010, Theodorides *et al*. [11] recommended that open operative procedures should be performed in all such patients.

We present an unusual case of an elderly woman with moderate arthritis who had a locked knee presenting as a patellar dislocation caused by an impaction fracture of the patella into the lateral femoral condyle. In this case report, we confirm the need for surgery to prevent relocking but demonstrate that such injuries can be treated by performing arthroscopy rather than an open surgical procedure. This point is particularly relevant because as population demographics change and such injuries become more common in patients with degenerative knees, short, minimally invasive procedures and reduced recovery times are important to preserving patients' mobility.

## Case presentation

An 88-year-old Caucasian woman was referred to our orthopedic unit following a simple trip on the stairs leading to a locked knee at an 80° angle. In the fall, the quadriceps muscles were forcefully contracted on her bent knee. Prior to the incident, she was independently mobile with the use of a stick. Her medical history included osteoarthritis affecting both knees and mild, generalized, right-sided weakness following a subdural hemorrhage secondary to an RTA. The physical examination of her right knee revealed a closed injury with minimal swelling. We noted a tender, inferiorly displaced patella. Her range of movement of the knee was 80°to 115° with a definite block to extension.

**Figure 1:**
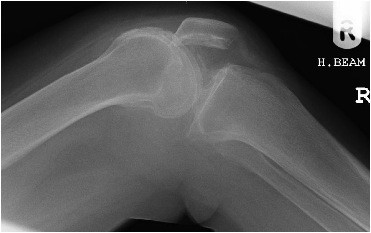
**Plain lateral view X-ray obtained at the time of admission**.

The initial plain radiographs of her flexed knee revealed inferior displacement of the patella (Figure 1).

**Figure 2:**
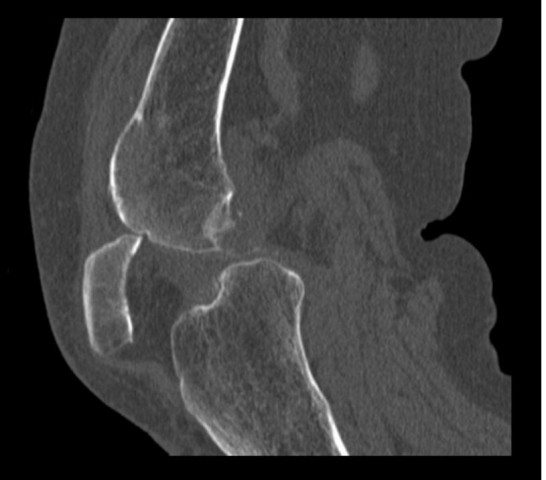
**Computed tomography sagittal image showing the superior pole of the patella impacted in the lateral condyle**.

Under patient sedation, the patient¹s patellar swelling was reduced by hyperflexing the knee, placing downward pressure on the inferior pole of the patella, and then slowly extending her leg. Her knee was then placed in a camp splint and allowed to mobilize. The following day the patient was able to raise and straighten her leg with a relatively pain-free range of movement of the knee and was able to mobilize independently on the ward. While in the hospital, she was unable to tolerate the splint and abandoned it. Her knee then locked again following flexion past 90°. A computed tomography scan of her right knee was performed, which showed a superior patellar osteophyte embedded in the lateral femoral condyle (Figure 2).

**Figure 3:**
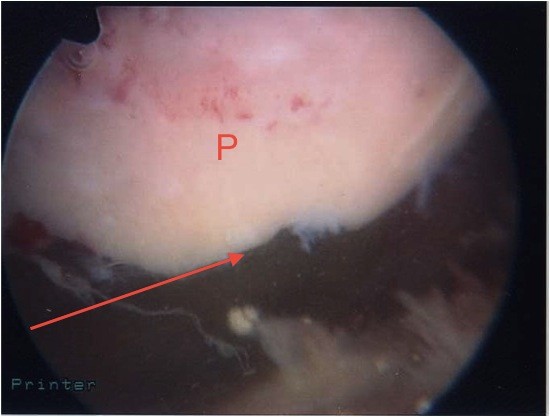
**View of the patellar osteophyte (arrow) during arthroscopy before debridement**. P indicates patella.

**Figure 4:**
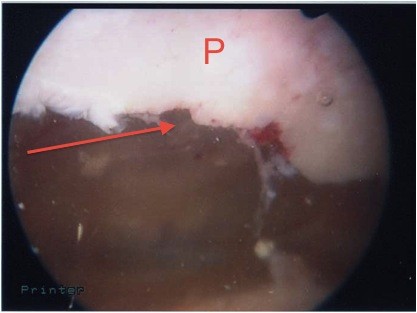
**View of patella after debridement (arrow)**. P indicates patella.

**Figure 5:**
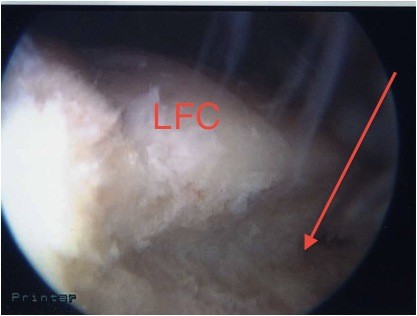
**View of the ridge on the lateral femoral condyle (LFC) during arthroscopy before debridement**. Arrow indicates ridge.

**Figure 6:**
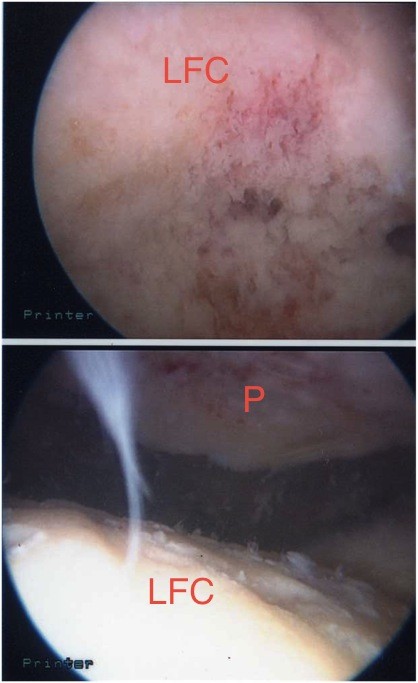
**View of the ridge on the lateral femoral condyle (LFC) during arthroscopy after debridement**. P indicates patella.

**Figure 7:**
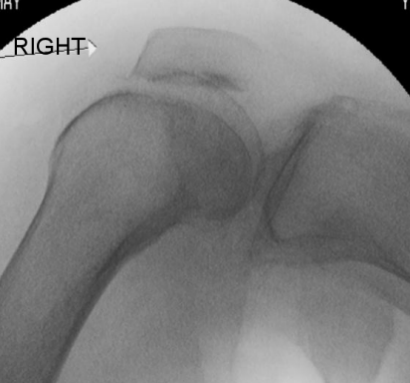
**Fluoroscopic images of the trimmed patella and the lateral femoral condyle showing no locking of the patella with the knee in flexion**.

The patient's knee was examined while she was under general anesthesia. When her knee was flexed past 90°, the patella locked into the lateral femoral condyle. The knee could be unlocked by slightly increasing flexion and applying pressure inferomedially while slowly extending the knee. Arthroscopy was performed using standard lateral and medial portals. This confirmed the presence of a deep ridge where the patellar osteophyte had become embedded into the arthritic lateral condyle, causing locking of the knee when flexed passed 90°. The rest of her knee had grade III-IV osteoarthritic changes. With a burr, the superior pole of the patella (Figures 3 and 4) was trimmed and the ridge on the lateral femoral condyle was smoothed (Figures 5 and 6). The results of this procedure were checked using a fluoroscopic image intensifier (Figure 7). When reexamined, the patella tracked smoothly over the lateral condyle without locking.

Post-operatively, the patient showed marked improvement in her symptoms. She was able to raise and straighten her leg and extend her right knee. Mobilization was possible without recurrence of her right knee locking, and her pre-injury mobility was regained within one week. In her last review at the clinic 12 weeks later, she was found to have retained her pre-injury mobility and was delighted with the outcome of her surgery.

## Discussion

There are a number of reports in the literature about arthritic and locked knees [1,6,9,10,12]. Simple manipulation and immobilization have been recommended for the management of elderly patients with patellar dislocations, as the mechanism of locking is understood to be osteophytes on the pole of the patella becoming entrapped in the inter-condylar notch [10]. In our patient, a patellar osteophyte was impacted into the lateral femoral condyle, causing locking of the knee.

Femoral condyle articular damage has been reported in younger patients with hemophilia who presented with locked knees and were treated with simple closed manipulation, but they had long, incomplete recovery times of up to one year [13]. There have also been reports of articular damage in elderly patients managed by performing open operative procedures, suggesting that locked knees in elderly patients are not benign [10,11].

The current recommendations in the literature for irreducible and recurrent dislocations are open reduction and exploration, but such procedures may lead to longer recovery times than that described in the present report [9,11]. Herein we describe management with arthroscopic procedures which allowed a short in-patient stay and a good, immediate return to pre-injury mobility within one week after surgery with minimal soft tissue disruption. To the best of our knowledge, this is the first report of patient with a locked knee with lateral condyle impaction fracture that was recognized as such and was managed successfully by performing arthroscopic surgery. The changing demographics of the population suggest the likelihood of an increase in such presentations.

## Conclusion

Locked knees require careful assessment, especially in the elderly. Impaction fractures should be considered in all rare cases of patellar dislocation, and we advocate arthroscopic assessment of the articular cartilage in such cases.

## Consent

Written informed consent was obtained from the patient for publication of this case report and any accompanying images. A copy of the written consent is available for review by the Editor-in-Chief of this journal.

## Competing interests

The authors declare that they have no competing interests.

## Authors' contributions

DB, SJR, KSF, and WS reviewed the literature and drafted the manuscript. SJG and DB reviewed the manuscript and supervised the conception and design of the report. All authors read and approved the final manuscript.
